# Supplementary material for: Time series modeling of cell cycle exit identifies Brd4 dependent regulation of cerebellar neurogenesis
Source: Nat Commun. 2019 Jul 10;10:3028. doi: 10.1038/s41467-019-10799-5 (PMC6620341; doi:10.1038/s41467-019-10799-5)
Supplement: Supplementary file 1 — Supplementary Information [file 41467_2019_10799_MOESM1_ESM.pdf]

Time series Modeling of Cell Cycle Exit Identifies Brd4 dependent Regulation of Cerebellar Neurogenesis

SUPPLEMENTARY INFORMATION

PENAS ET AL.

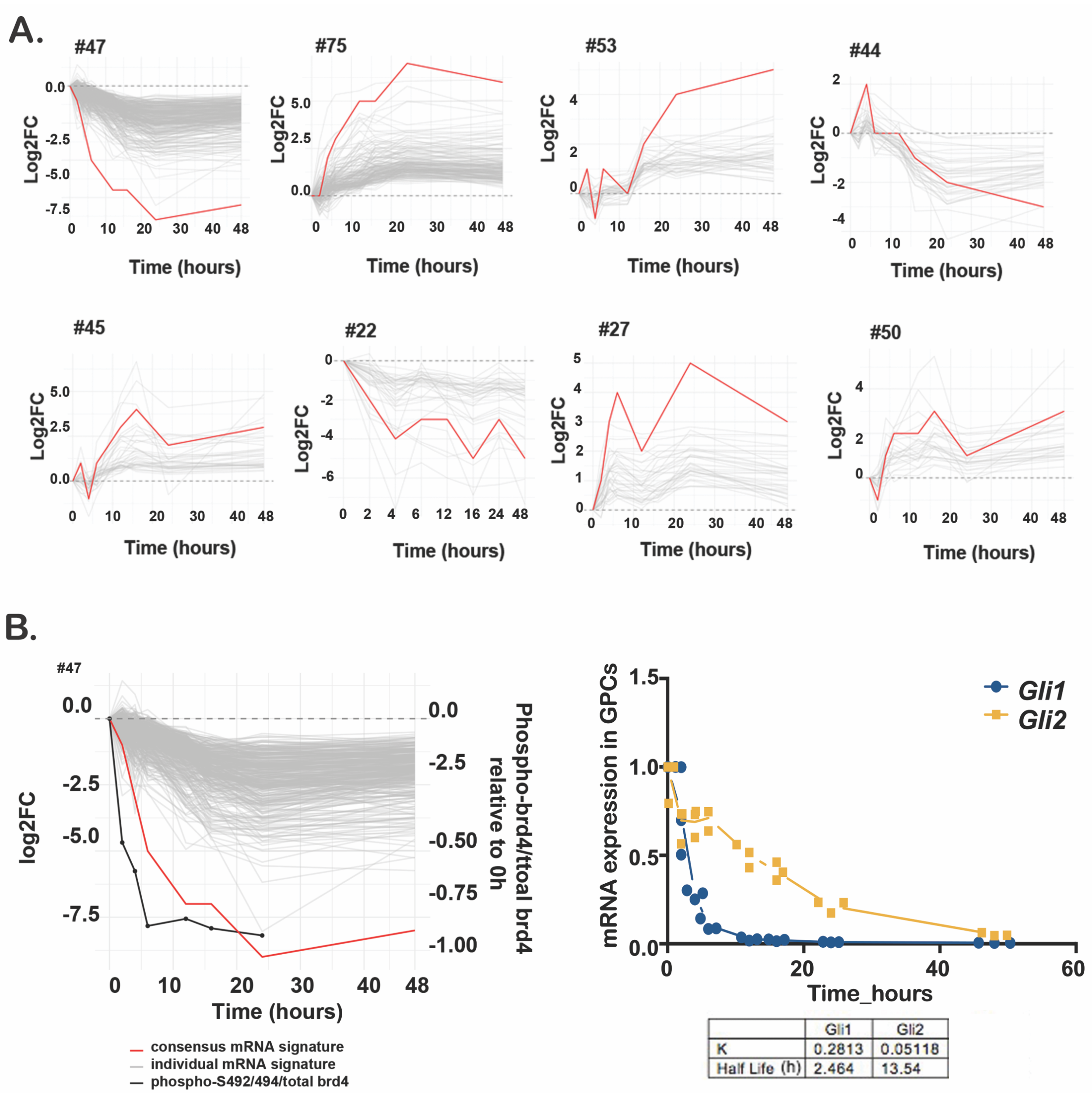

Figure 1. Time series modelling of cell cycle exit in GCPs demonstrates that phospho-Brd4 clusters with *Gli1* and *Gli2*. A) Short time-series expression miner was used to cluster mRNA expression profiles during GCP cell cycle exit. All clusters with significant differential gene expression are shown. Dotted line indicates no expression change. Clustered genes that appear above the dotted line show increased expression, and clustered genes that appear below decreased expression relative to 0 h. **B)** Left- Phospho-Brd4 S492/494 kinetics are overlain with cluster #47, which contains the *Gli1* and *Gli2* signature. Right- Corroboration of mRNA levels of *Gli1* and *Gli2,* by qPCR, are shown over the time course. Source data can be found in source data graphs under tab for supplementary figure 1B.

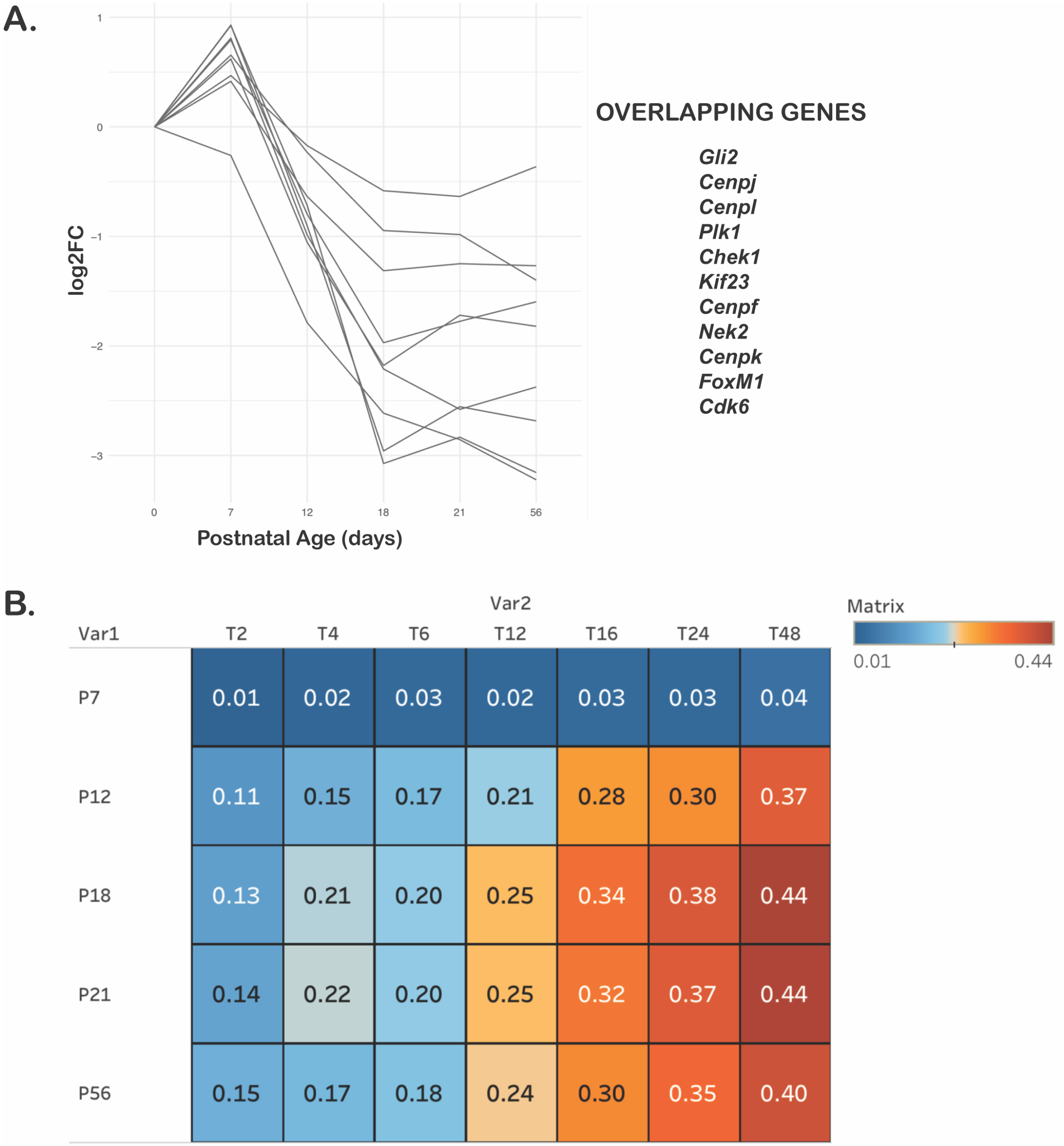

Figure 2. Time series modelling of cell cycle gene expression during cerebellum development correlates with modelling during GCP cell cycle exit. Gene expression data from Zhu et al.[1] over postnatal (P0-P56) development were clustered as shown in Supplementary Figure 1. **A)** A subset of cluster #47 genes are plotted and normalized to expression levels at P0. For most genes in this cluster there is a relative increase of expression at P7, at a time when GCPs are actively proliferating, followed by a persistent decrease in expression that persists throughout development. **B)** Pairwise comparison of the percentage of DEG overlap between the two datasets. Columns indicate the hours post plating (T2-T48) and rows indicate the postnatal age (P7-P56) and samples were compared to T0 and P0 respectively.

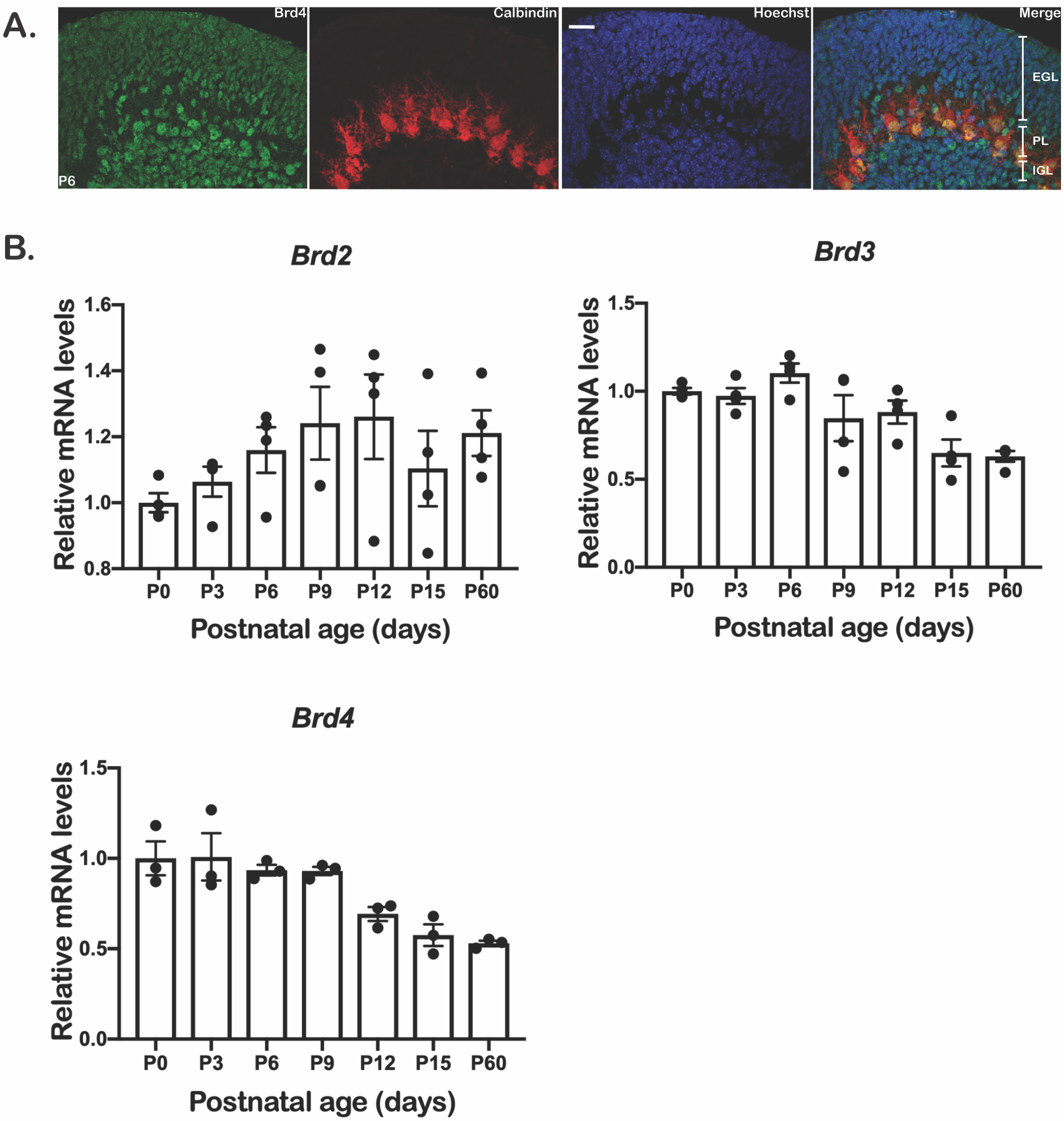

Figure 3. BET proteins are expressed in the cerebellum during development.
A) Immunostaining of sagittal cerebella from P6 mice for Brd4, calbindin (Purkinje cells) and DNA (Hoechst). Scale=20μM. EGL= external granule layer, ML= molecular layer, PL= Purkinje cell layer, IGL= internal granule layer. **B)** Relative expression of *Brd2, Brd3*, *and Brd4* in whole cerebellum over the course of development. RNA was extracted from whole cerebellum of CD1 mice at the developmental ages indicated. qRT-PCR was performed and normalized to *Gapdh*. Results are shown as the average values of three cerebella per age and are represented as the mean ± SEM. Source data can be found in source data graphs under tab for supplementary figure 3B.

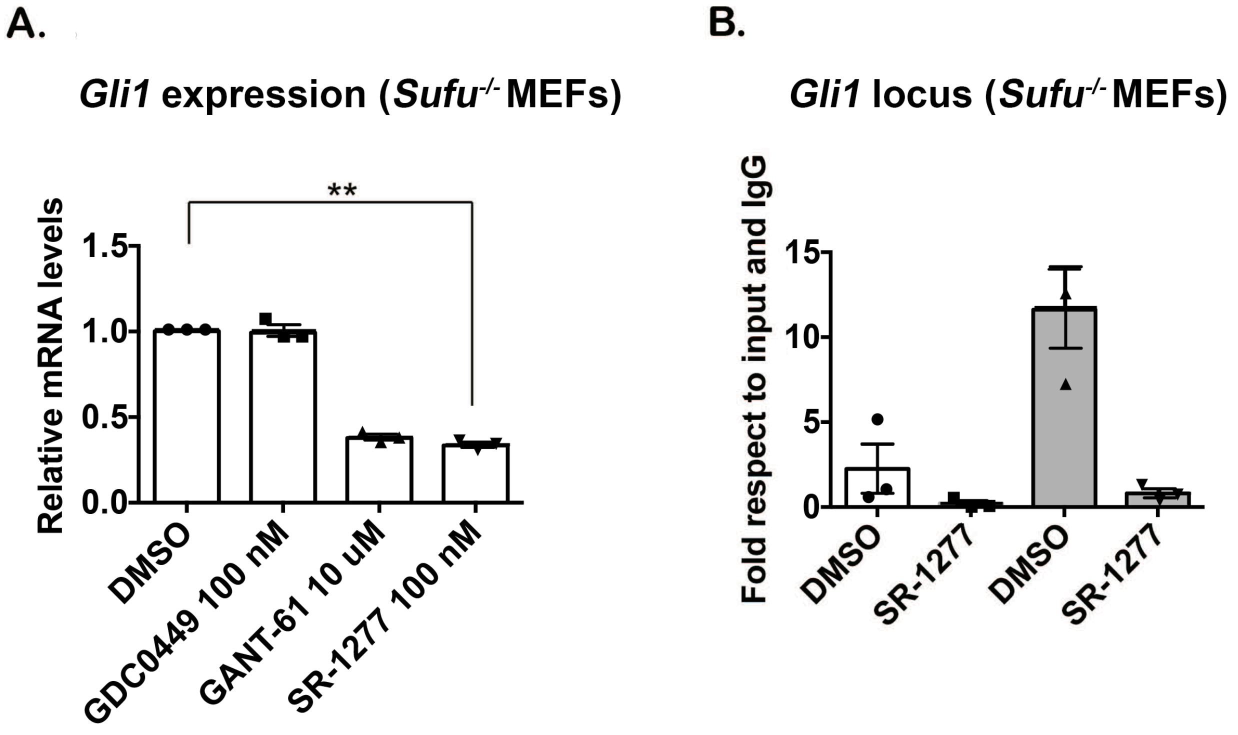

Figure 4. CK1δ inhibition reduces *Gli1* mRNA and Brd4 binding to the *Gli1* locus downstream of the SHH effector Smoothened A) CK1δ inhibition reduced *Gli1* mRNA expression Sufu$^{-/-}$ MEFs. Sufu$^{-/-}$ MEFs were treated for 24 h with SR-1277 (CK1δ inhibitor, 100 nM), GDC0449[2] (Smo inhibitor, 100 nM) and GANT-61[3] (Gli inhibitor, 10 μM); The latter two were used as negative and positive controls, respectively. mRNA was amplified by qRT-PCR and fold change in gene expression was determined by normalizing to *gapdh* values relative to control samples. Results shown are averages of three independent experiments and are represented as the mean ± SEM. A one-way ANOVA followed by Tukey's multiple comparison testing was performed (**$p < 0.01$). **B)** CK1δ inhibition reduces Brd4 binding to the *Gli1* locus in Sufu$^{-/-}$ MEFs. Sufu$^{-/-}$ MEFs were incubated with SR-1277 or DMSO and the levels of Brd4 bound to the *Gli1* locus were determined as described. Source data can be found in source data graphs under tabs for supplementary figure 4A and 4B.

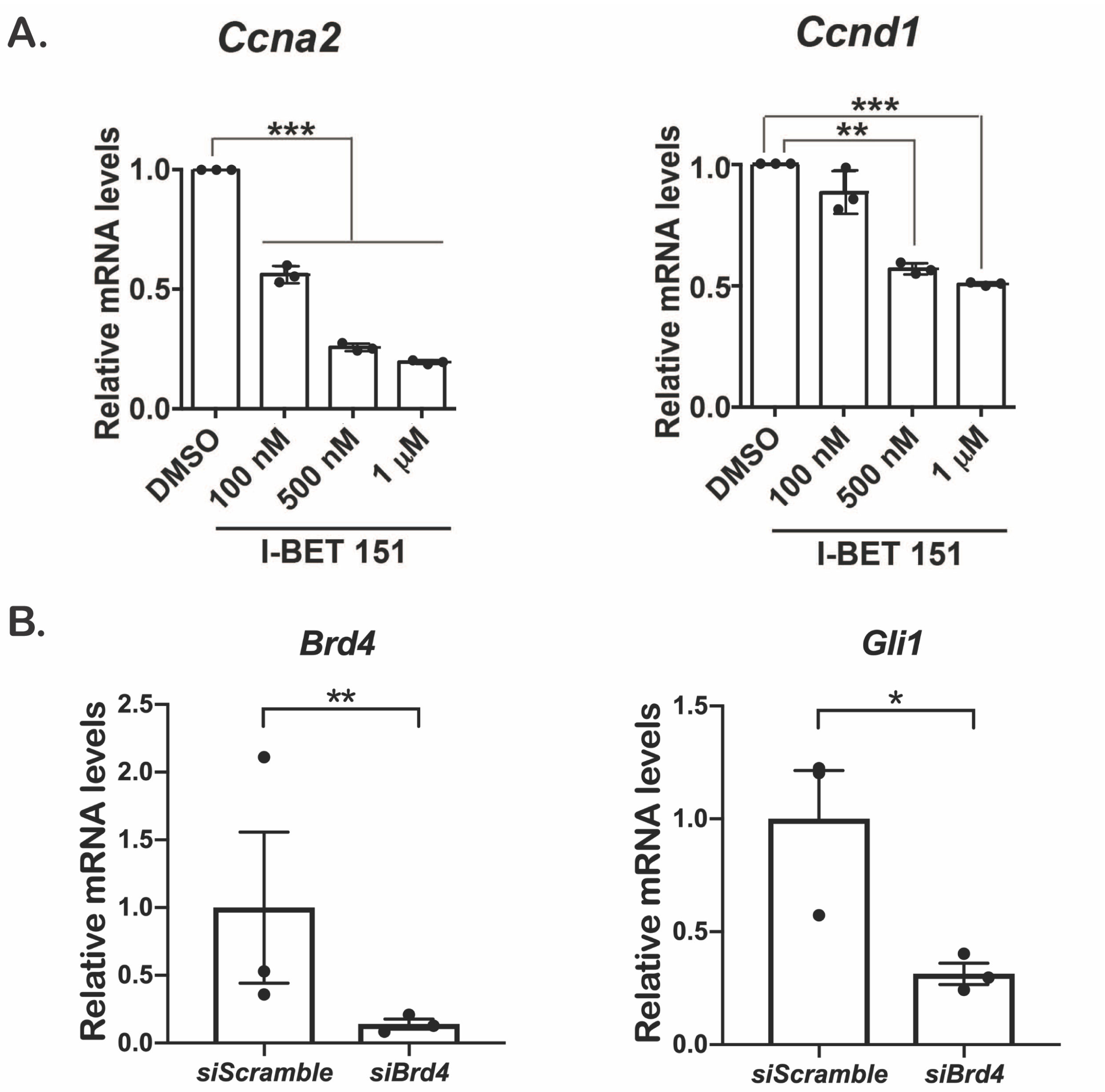

Figure 5. BET bromodomain protein inhibition reduces expression of cyclins and Brd4 knockdown reduces *Gli1* expression in GCPs. A) BET protein inhibition reduces GCP *Cyclin a2 (Ccna2) and Cyclin d1(Ccnd1)* mRNA levels *in vitro*. GCPs were purified from P6 CD1 mice and incubated with the indicated concentrations of I-BET151 or DMSO control for 24 h. qRT-PCR was performed and normalized to *Gapdh*. **B)** GCPs were purified from P6 CD1 mice for Brd4 knockdown using an SMARTpool siRNA and scramble control. Brd4 knockdown produced an 80% of Brd4 knockdown, which resulted in a 75% decrease of *Gli1* mRNA expression. qRT-PCR was performed and normalized to *Gapdh*. Results shown are averages of three independent experiments and are represented as the mean ± SEM. A one-way ANOVA followed by Tukey's multiple comparison testing (5A) or a two-tailed t-test (5B) was performed (*p < 0.05, **p < 0.01, *** p < 0.001). Source data can be found in source data graphs under tabs for supplementary figure 5A and 5B.

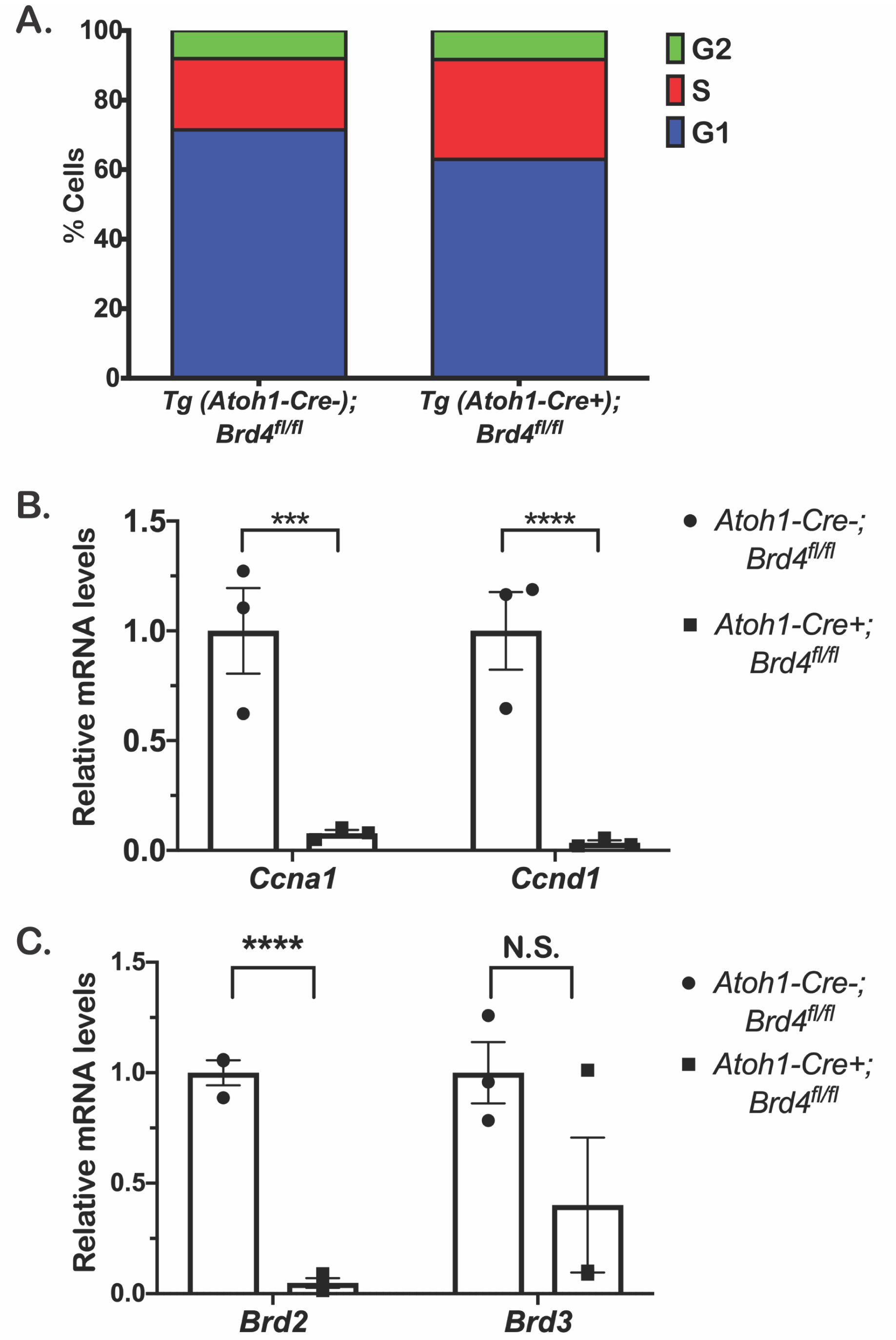

Figure 6. Conditional deletion of Brd4 in purified GCPs alters cell cycle kinetics and reduces expression of cyclins and BET proteins. A) PI-FACS analysis was performed on GCPs purified from P8 Tg (*Atoh1-Cre+);Brd4$^{fl/fl}$* and Tg (*Atoh1-Cre-);Brd4$^{fl/fl}$* mice. Flow Jo software was then used to assess the percentage of cells in the G1, S, or G2/M phase. **B-C)** GCPs from Tg (*Atoh1-Cre+);Brd4$^{fl/fl}$* mice express less *Cyclin a1 (Ccna1)*, *Cyclin d1 (Ccnd1)*, and *Brd2* than Tg (*Atoh1-Cre-);Brd4$^{fl/fl}$* mice. RNA was extracted from isolated GCPs from P8 mice, and qRT-PCR was performed and normalized to *Gapdh*. Results are the average of three independent experiments and are represented as the mean ± SEM. Two-tailed t-tests were performed (***p < 0.001, ****p< 0.0001, N.S. no significance). Source data can be found in source data graphs under tabs for supplementary figure 6B and 6C.

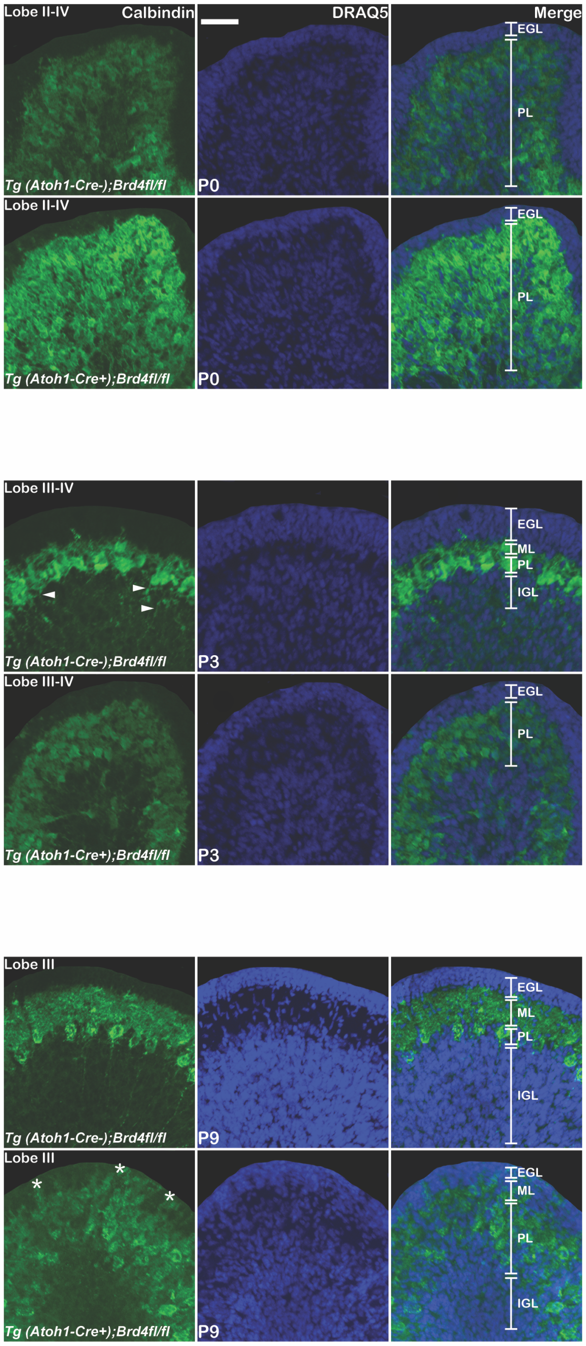

Figure 7. Conditional deletion of Brd4 in the developing anterior cerebellum induces persistent cerebellar deficits. Brd4 loss in GCPs disrupts Purkinje cell monolayer development and neurite outgrowth. Confocal images are from the mid vermis anterior cerebellum of P0, P3 and P9 Tg (*Atoh1-Cre+);Brd4*$^{fl/fl}$ and Tg (*Atoh1-Cre-);Brd4*$^{fl/fl}$ mice. Images approximate the same lobe at each developmental time point and are indicate in the figure. Disruption to the Purkinje cell layer is noted beginning at P3. Calbindin is a marker for Purkinje cells, and DRAQ5 is a DNA marker. Scale=50μM. EGL= external granule layer, ML= molecular layer, PL= Purkinje cell layer, IGL= internal granule layer. Arrowheads indicate neurites from Purkinje cells that are present in the Tg (*Atoh1-Cre-);Brd4*$^{fl/fl}$ cerebellum that are absent in the Tg (*Atoh1-Cre+);Brd4*$^{fl/fl}$ cerebellum. Asterisks indicate Purkinje cell neurites that are abnormally present in the the EGL of the Tg (*Atoh1-Cre+);Brd4*$^{fl/fl}$ cerebellum vs. the Tg (*Atoh1-Cre-);Brd4*$^{fl/fl}$ cerebellum.

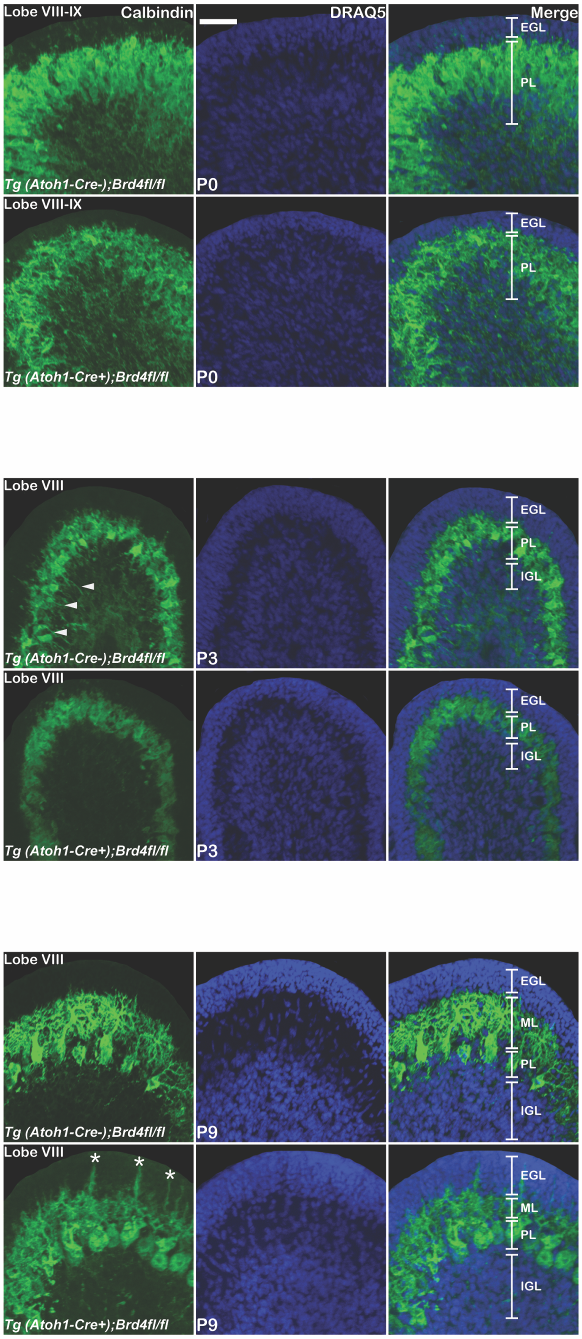

Figure 8. Conditional deletion of Brd4 in the developing posterior cerebellum induces persistent cerebellar deficits. Brd4 loss in GCPs disrupts Purkinje cell neurite outgrowth. Confocal images are from the mid vermis posterior cerebellum of P0, P3 and P9 Tg (*Atoh1-Cre+);Brd4*$^{fl/fl}$ and Tg (*Atoh1-Cre-);Brd4*$^{fl/fl}$ mice. Images approximate the same lobe at each developmental time point and are indicate in the figure. Calbindin is a marker for Purkinje cells, and DRAQ5 is a DNA marker. Scale=50μM. EGL= external granule layer, ML= molecular layer, PL= Purkinje cell layer, IGL= internal granule layer. Arrowheads indicate neurites from Purkinje cells that are present in the Tg (*Atoh1-Cre-);Brd4*$^{fl/fl}$ cerebellum that are absent in the Tg (*Atoh1-Cre+);Brd4*$^{fl/fl}$ cerebellum. Asterisks indicate Purkinje cell neurites that are abnormally present in the the EGL of the Tg (*Atoh1-Cre+);Brd4*$^{fl/fl}$ cerebellum vs. the Tg (*Atoh1-Cre-);Brd4*$^{fl/fl}$ cerebellum.

Supplementary File 1. Genes and GO terms associated with clusters in Figure 1D, Supplementary Figure 1.

Source Data Figures 1-5. Each figure contains uncropped blots and gels presented in each sub-figure. Red box highlighted region indicates portion of block used in figure.

Source Data Graphs. Spreadsheet containing raw numbers presented in graphs. Each tab corresponds to graph(s) presented in each sub-figure or supplementary sub-figure.

References

1. Zhu, X. *et al.* Role of Tet1/3 Genes and Chromatin Remodeling Genes in Cerebellar Circuit Formation. *Neuron* **89**, 100-112 (2016).
2. Robarge, K.D. *et al.* GDC-0449-a potent inhibitor of the hedgehog pathway. *Bioorg Med Chem Lett* **19**, 5576-5581 (2009).
3. Desch, P. *et al.* Inhibition of GLI, but not Smoothened, induces apoptosis in chronic lymphocytic leukemia cells. *Oncogene* **29**, 4885-4895 (2010).
