# Supplementary material for: Time series modeling of cell cycle exit identifies Brd4 dependent regulation of cerebellar neurogenesis
Source: Nat Commun. 2019 Jul 10;10:3028. doi: 10.1038/s41467-019-10799-5 (PMC6620341; doi:10.1038/s41467-019-10799-5)
Supplement: Supplementary file 3 — Reporting Summary [file 41467_2019_10799_MOESM3_ESM.pdf]

nature research

Corresponding author(s): Nagi Ayad

Last updated by author(s): Apr 24, 2019

Reporting Summary

Nature Research wishes to improve the reproducibility of the work that we publish. This form provides structure for consistency and transparency in reporting. For further information on Nature Research policies, see Authors & Referees and the Editorial Policy Checklist.

Statistics

For all statistical analyses, confirm that the following items are present in the figure legend, table legend, main text, or Methods section.

n/a	Confirmed	
☐	☒	The exact sample size (*n*) for each experimental group/condition, given as a discrete number and unit of measurement
☐	☒	A statement on whether measurements were taken from distinct samples or whether the same sample was measured repeatedly
☐	☒	The statistical test(s) used AND whether they are one- or two-sided<br>*Only common tests should be described solely by name; describe more complex techniques in the Methods section.*
☐	☒	A description of all covariates tested
☐	☒	A description of any assumptions or corrections, such as tests of normality and adjustment for multiple comparisons
☐	☒	A full description of the statistical parameters including central tendency (e.g. means) or other basic estimates (e.g. regression coefficient) AND variation (e.g. standard deviation) or associated estimates of uncertainty (e.g. confidence intervals)
☐	☒	For null hypothesis testing, the test statistic (e.g. *F*, *t*, *r*) with confidence intervals, effect sizes, degrees of freedom and *P* value noted<br>*Give P values as exact values whenever suitable.*
☒	☐	For Bayesian analysis, information on the choice of priors and Markov chain Monte Carlo settings
☒	☐	For hierarchical and complex designs, identification of the appropriate level for tests and full reporting of outcomes
☒	☐	Estimates of effect sizes (e.g. Cohen's *d*, Pearson's *r*), indicating how they were calculated

Our web collection on statistics for biologists contains articles on many of the points above.

Software and code

Policy information about availability of computer code

Data collection	ThermoScientific NanoDrop and Agilent Bioanalyzer
Data analysis	FastQC and TopHatv.2.1 with CuffDiff 2.2; Functional Annotation Analysis using DAVID; Short Time Series Expression Miner (STEM); Prism (Graphpad)

For manuscripts utilizing custom algorithms or software that are central to the research but not yet described in published literature, software must be made available to editors/reviewers. We strongly encourage code deposition in a community repository (e.g. GitHub). See the Nature Research guidelines for submitting code & software for further information.

Data

Policy information about availability of data

All manuscripts must include a data availability statement. This statement should provide the following information, where applicable:

- Accession codes, unique identifiers, or web links for publicly available datasets
- A list of figures that have associated raw data
- A description of any restrictions on data availability

NCBI accession number SRP146255

Field-specific reporting

Please select the one below that is the best fit for your research. If you are not sure, read the appropriate sections before making your selection.

☒ Life sciences ☐ Behavioural & social sciences ☐ Ecological, evolutionary & environmental sciences

Life sciences study design

All studies must disclose on these points even when the disclosure is negative.

Sample size	Sample size was determined based on results from pilot data.
Data exclusions	No data were excluded
Replication	Verification of hypothesized functional gene effects from sequencing experiments was performed by in vitro studies
Randomization	Random
Blinding	Investigators were blinded to group assignment

Reporting for specific materials, systems and methods

We require information from authors about some types of materials, experimental systems and methods used in many studies. Here, indicate whether each material, system or method listed is relevant to your study. If you are not sure if a list item applies to your research, read the appropriate section before selecting a response.

Materials & experimental systems

n/a	Involved in the study	
☐	☒	Antibodies
☒	☐	Eukaryotic cell lines
☒	☐	Palaeontology
☐	☒	Animals and other organisms
☒	☐	Human research participants
☒	☐	Clinical data

Methods

n/a	Involved in the study	
☒	☐	ChIP-seq
☐	☒	Flow cytometry
☒	☐	MRI-based neuroimaging

Antibodies

Antibodies used	Mouse anti-CK1δ (C-8) (1/1000, Santa Cruz Biotechnology, sc-55553), goat anti-CK1δ (N-19) (1/500, sc-6475, Santa Cruz Biotechnology), rabbit-anti C-terminal Brd4 (1/2000)6, rabbit anti-Brd4 (1/1000, A301-985A50, Bethyl Laboratories Inc.), rabbit anti-phospho-Brd4 492/494 (1/2000, ABE1453, Millipore), mouse anti-Gapdh (1/5000, NB300-221, Novus Biolechne), mouse anti-Flag (1/5000, A8592, Sigma) and rabbit anti-cyclin D1 (1/1000, ab134175, AbCam).Rabbit anti-Brd4 (1/1000, Bethyl Laboratories Inc.), mouse or rabbit anti-calbindin (1/1000, Swant300, CB38), and mouse anti-Tag1 (1/1, generously provided by M.E. Hatten).
Validation	Antibodies were previously validated by manufacturers. In the case of anti-Tag1, the antibody was validated in M.E. Hatten laboratory .

Animals and other organisms

Policy information about studies involving animals; ARRIVE guidelines recommended for reporting animal research

Laboratory animals	Male and female pups from postnatal day 6-8 from CD1 and Tg (Atoh1-Cre+);CK1δfl/fl strains were used for in vitro studies. Male and female pups from postnatal day 6-8 from Tg (Atoh1-cre)+/-;Brd4fl/fl and Tg (Atoh1-cre)-/-;Brd4fl/fl strains were used for in vitro studies.
Wild animals	No wild animals were used in this study.
Field-collected samples	N/A
Ethics oversight	IACUC committee at the University of Miami approved this protocol.

Note that full information on the approval of the study protocol must also be provided in the manuscript.

Flow Cytometry

Plots

Confirm that:

☒ The axis labels state the marker and fluorochrome used (e.g. CD4-FITC).

☐ The axis scales are clearly visible. Include numbers along axes only for bottom left plot of group (a 'group' is an analysis of identical markers).

☐ All plots are contour plots with outliers or pseudocolor plots.

☒ A numerical value for number of cells or percentage (with statistics) is provided.

Methodology

Sample preparation	GCPS were removed from dishes, washed with PBS and 1% BSA, and fixed with 10 % ethanol in PBS overnight at 4 °C. GCPs were then stained with 69 µM propidium iodide in 38 µM sodium citrate buffer and 1 µM RNAse A at 37 ºC for 30 minutes.
Instrument	fluorescence- activated cell sorting device (LSRII, Becton Dickinson)
Software	FlowJo software
Cell population abundance	The granule cell population obtained was 99% pure, as described before (Hatten, 1985)
Gating strategy	N/A

☐ Tick this box to confirm that a figure exemplifying the gating strategy is provided in the Supplementary Information.
