# Supplementary material for: Time series modeling of cell cycle exit identifies Brd4 dependent regulation of cerebellar neurogenesis
Source: Nat Commun. 2019 Jul 10;10:3028. doi: 10.1038/s41467-019-10799-5 (PMC6620341; doi:10.1038/s41467-019-10799-5)
Supplement: Supplementary file 4 — Description of Additional Supplementary Information [file 41467_2019_10799_MOESM4_ESM.pdf]

Description of Additional Supplementary Files

File Name: Supplementary Data 1
Description: Genes and GO terms associated with clusters in Figure 1D, Supplementary Figure 1.

File Name: Supplementary Movie 1
Description: **Representative ledge test for Tg (Atoh1-cre+);Brd4$^{fl/fl}$ and Tg (Atoh1- cre-);Brd4$^{fl/fl}$ mice.** The ledge test examines animal balance. The goal of the test is for the mouse to walk on the cage ledge then return to the cage floor. Mouse genotype status is indicated in the top of the screen as each mouse is performing the test.

File Name: Supplementary Movie 2
Description: **Representative gait test for Tg (Atoh1-cre+);Brd4$^{fl/fl}$ and Tg (Atoh1-cre-);Brd4$^{fl/fl}$ mice.** The gait test examines motor coordination. Mouse genotype status is indicated on the bottom of the screen as each mouse moves to the camera foreground. Note the tremor in the Tg (Atoh1- cre+);Brd4$^{fl/fl}$ versus Tg (Atoh1-cre-);Brd4$^{fl/fl}$.

File Name: Supplementary Movie 3
Description: **Representative rotarod test for Tg (Atoh1-cre+);Brd4$^{fl/fl}$ and Tg (Atoh1- cre-);Brd4$^{fl/fl}$ mice.** Mouse genotype status is indicated under each mouse during the duration of the testing. Note that the video was taken after five days of training.
