# Supplementary material for: Time series modeling of cell cycle exit identifies Brd4 dependent regulation of cerebellar neurogenesis
Source: Nat Commun. 2019 Jul 10;10:3028. doi: 10.1038/s41467-019-10799-5 (PMC6620341; doi:10.1038/s41467-019-10799-5)
Supplement: Supplementary file 9 — Source data [file 41467_2019_10799_MOESM9_ESM.pdf]

SOURCE DATA

Penas et al.

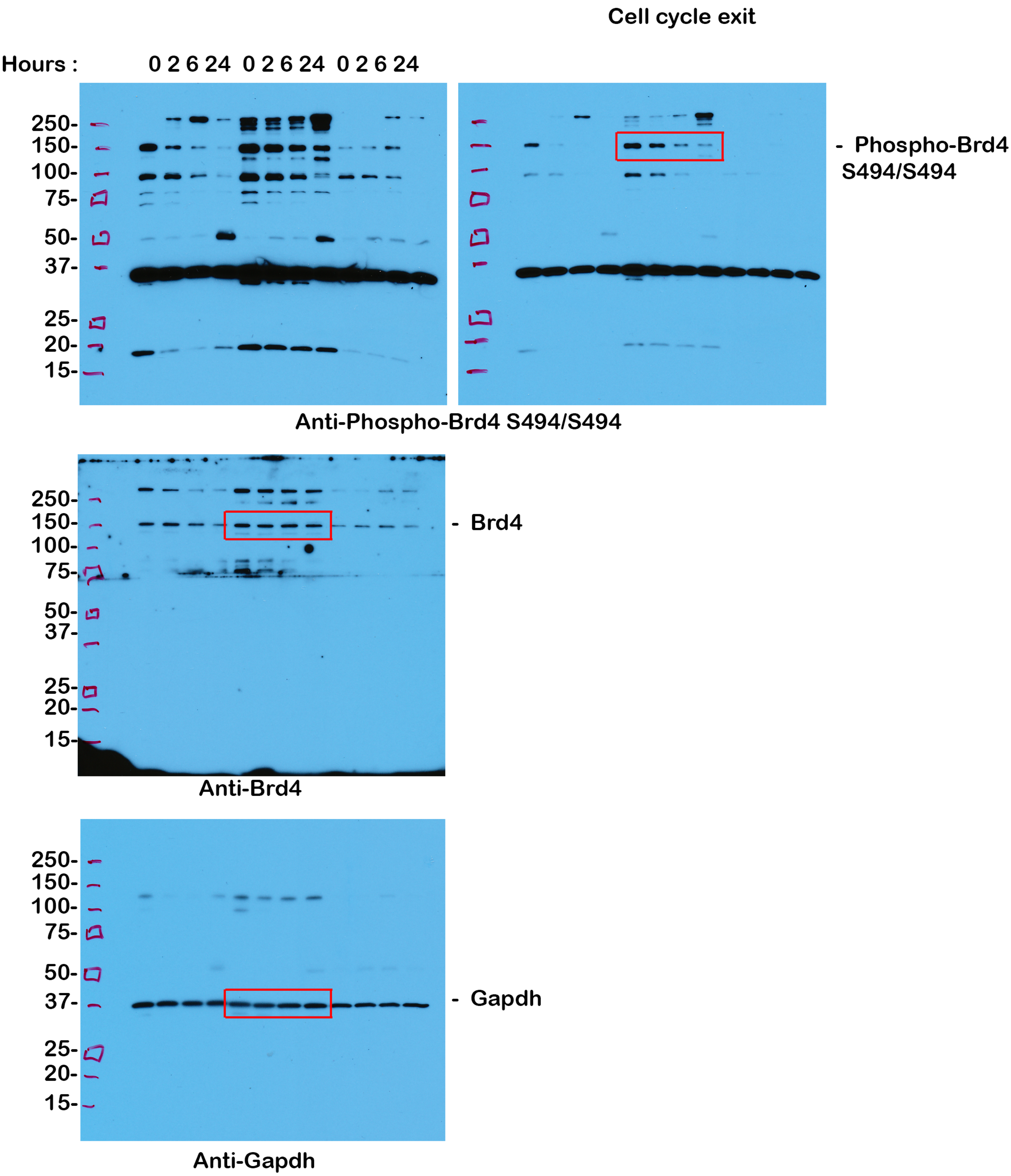

SOURCE DATA FIGURE 1

A.

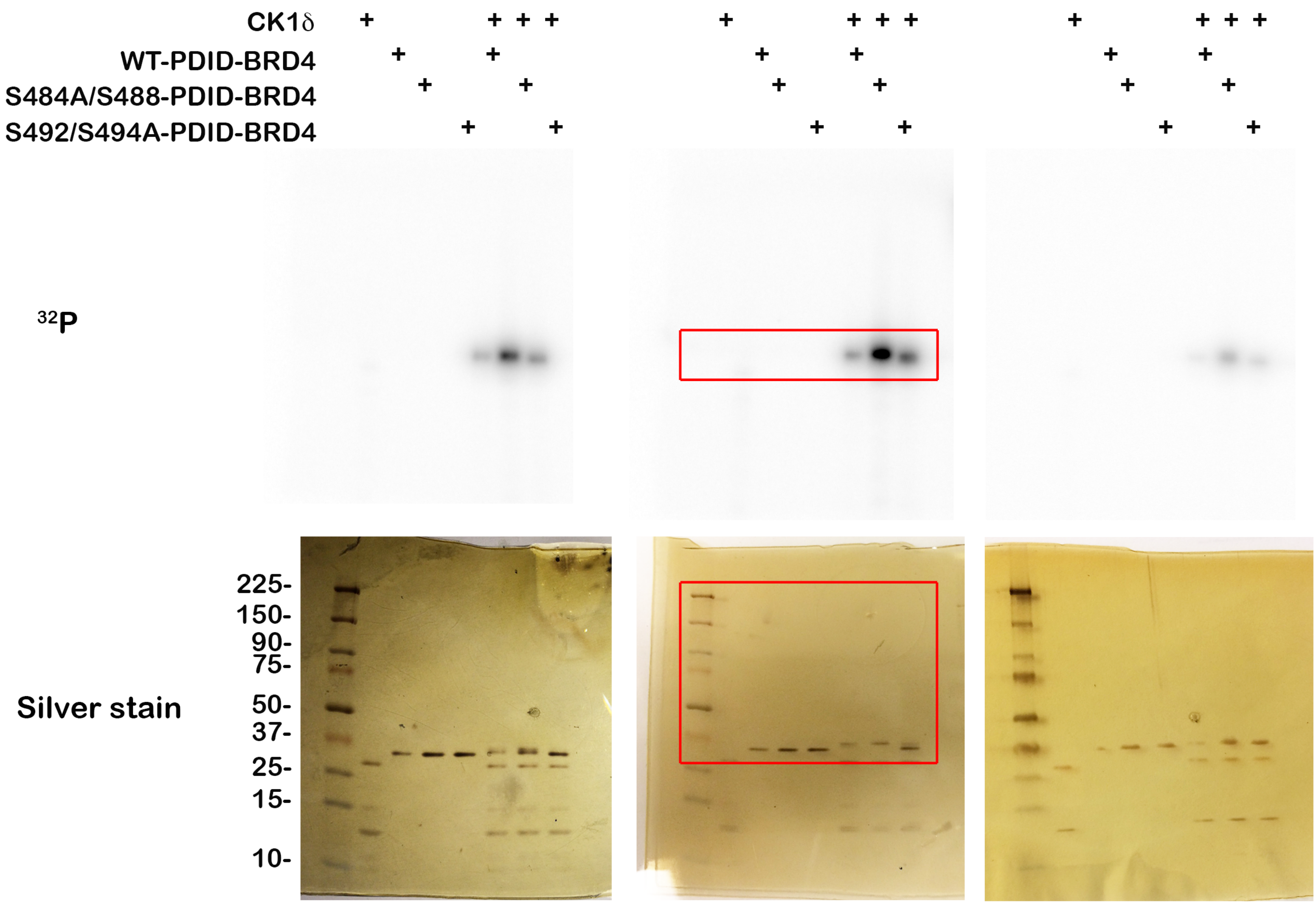

B.

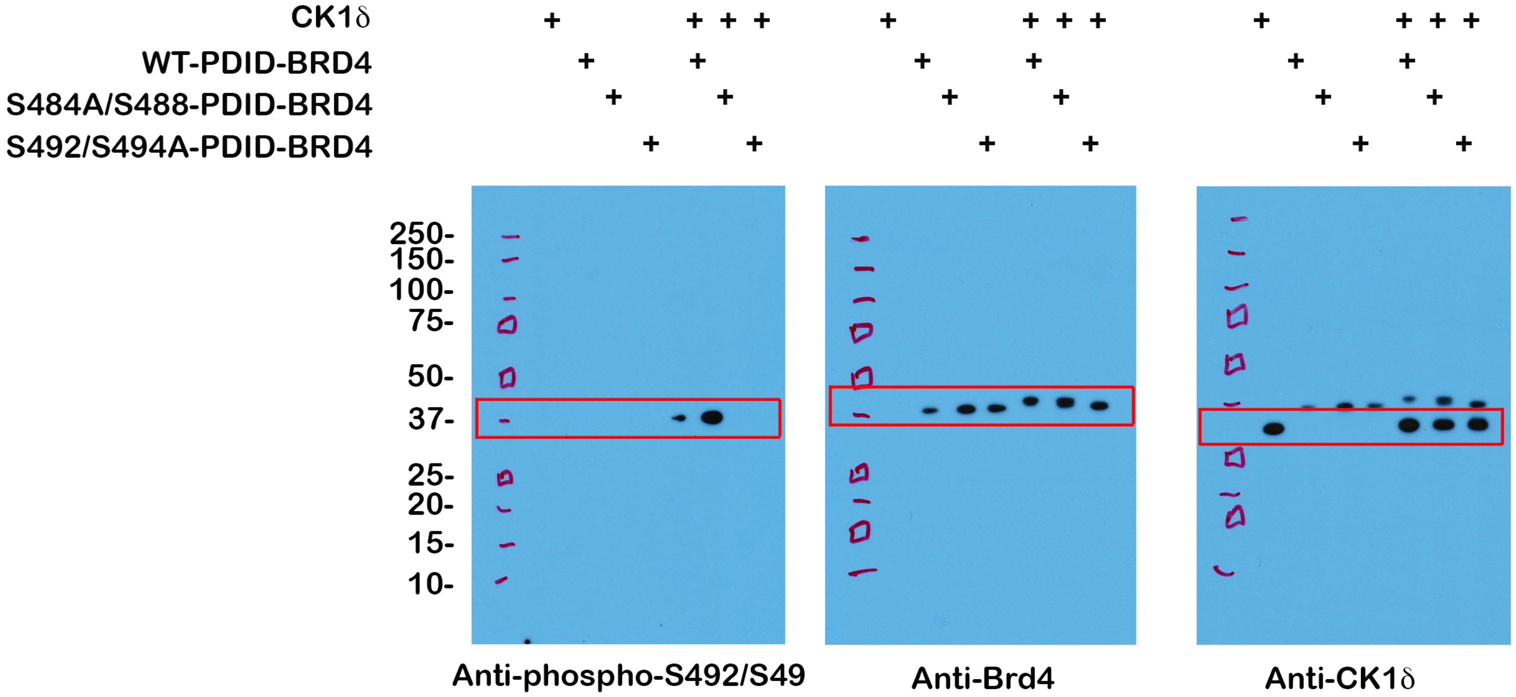

SOURCE DATA FIGURE 2

GCPs

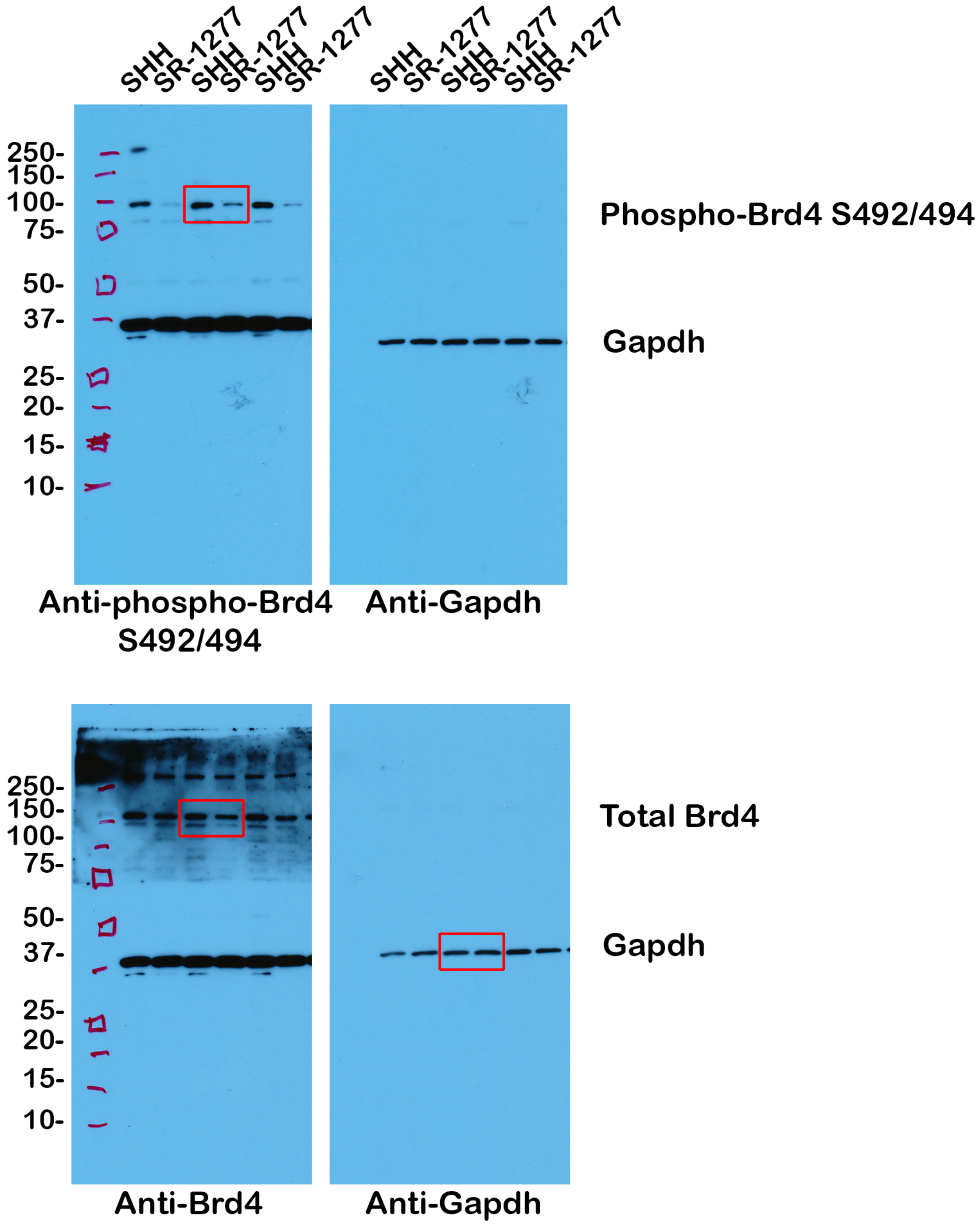

SOURCE DATA FIGURE 3

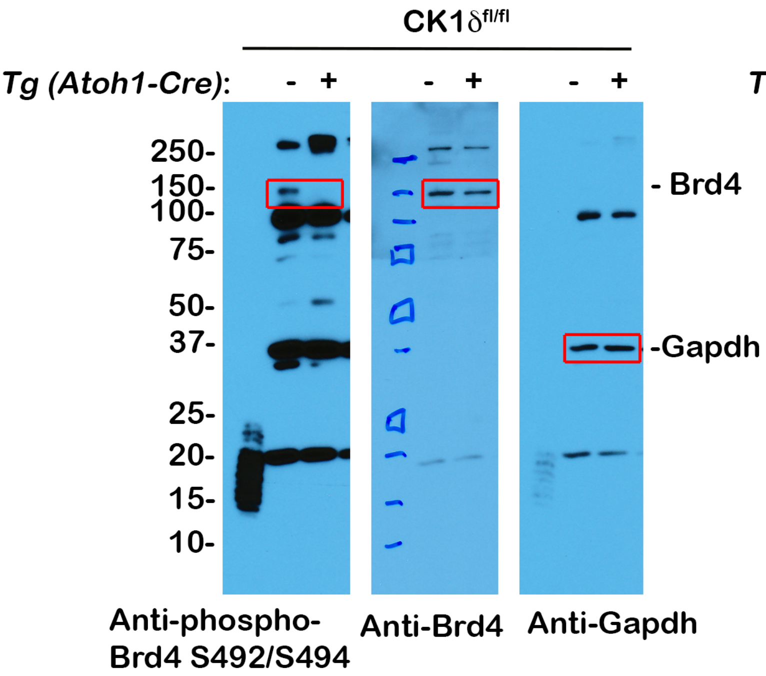

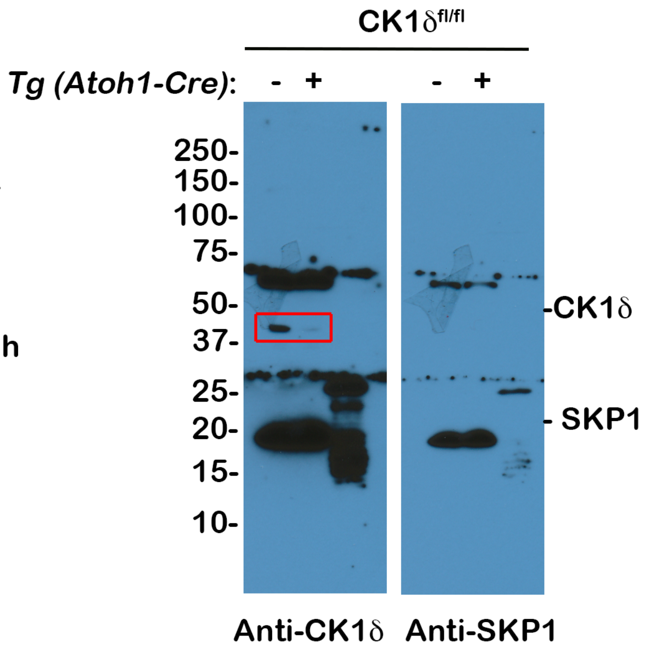

SOURCE DATA FIGURE 4

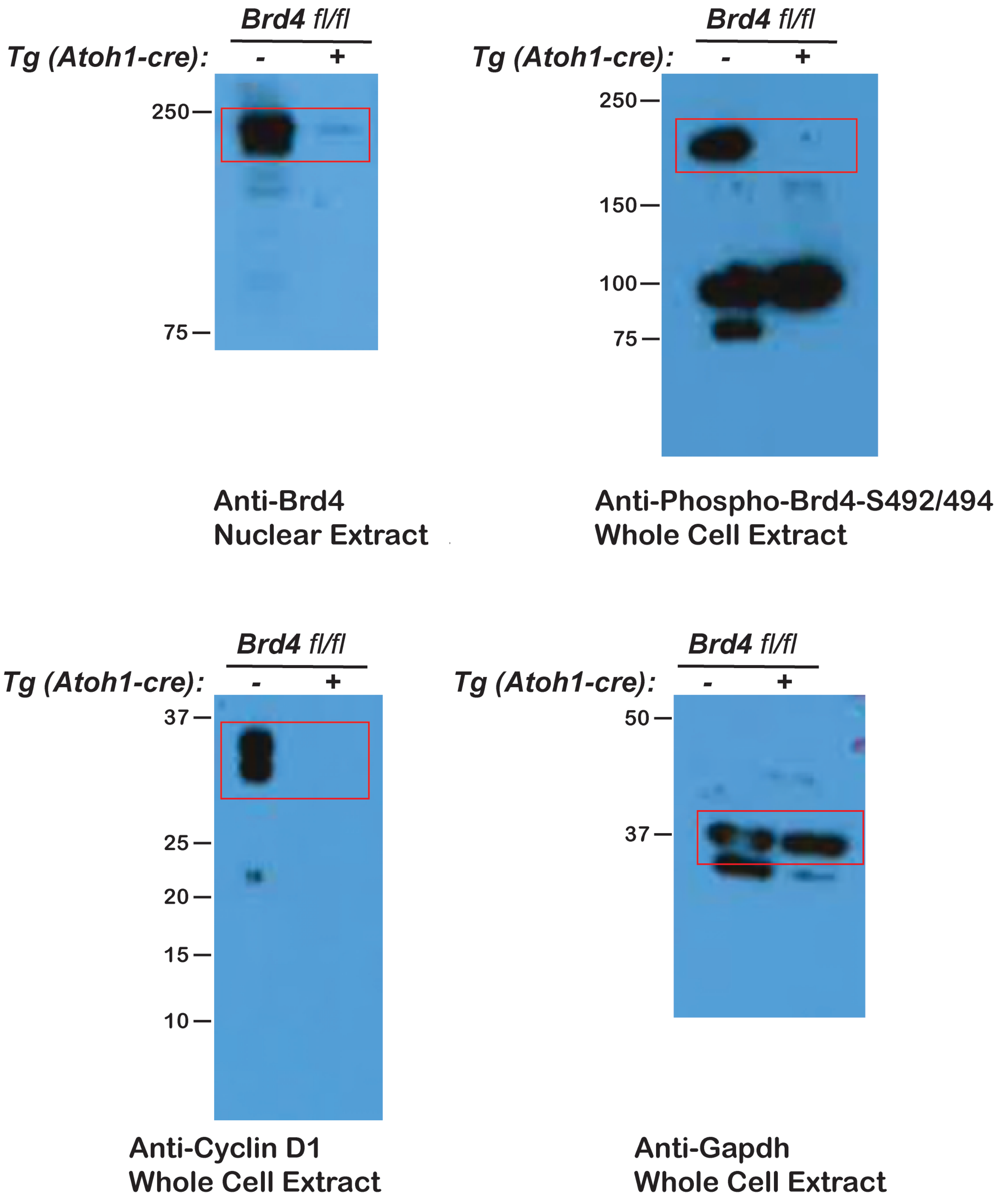

SOURCE DATA FIGURE 5
